# Multiple Changes in Peptide and Lipid Expression Associated with Regeneration in the Nervous System of the Medicinal Leech

**DOI:** 10.1371/journal.pone.0018359

**Published:** 2011-04-22

**Authors:** Céline Meriaux, Karim Arafah, Aurélie Tasiemski, Maxence Wisztorski, Jocelyne Bruand, Céline Boidin-Wichlacz, Annie Desmons, Delphine Debois, Olivier Laprévote, Alain Brunelle, Terry Gaasterland, Eduardo Macagno, Isabelle Fournier, Michel Salzet

**Affiliations:** 1 Université Lille Nord de France, Laboratoire de Spectrométrie de Masse Biologique Fondamentale et Appliquée (FABMS), EA 4550, Université Lille 1, Villeneuve d'Ascq, France; 2 Division of Biological Sciences, University of California San Diego, La Jolla, California, United States of America; 3 Institut de Chimie des Substances Naturelles, Centre de Recherche de Gif, Gif-sur-Yvette, France; 4 Chimie Toxicologie Analytique et Cellulaire, Faculté des Sciences Pharmaceutiques et Biologiques, Université Paris Descartes, Paris, France; 5 Marine Biology Research Division, Scripps Institution of Oceanography, Division of Biological Sciences, Institute of Genomic Medicine, University of California San Diego, La Jolla, California, United States of America; University of South Florida College of Medicine, United States of America

## Abstract

**Background:**

The adult medicinal leech central nervous system (CNS) is capable of regenerating specific synaptic circuitry after a mechanical lesion, displaying evidence of anatomical repair within a few days and functional recovery within a few weeks. In the present work, spatiotemporal changes in molecular distributions during this phenomenon are explored. Moreover, the hypothesis that neural regeneration involves some molecular factors initially employed during embryonic neural development is tested.

**Results:**

Imaging mass spectrometry coupled to peptidomic and lipidomic methodologies allowed the selection of molecules whose spatiotemporal pattern of expression was of potential interest. The identification of peptides was aided by comparing MS/MS spectra obtained for the peptidome extracted from embryonic and adult tissues to leech transcriptome and genome databases. Through the parallel use of a classical lipidomic approach and secondary ion mass spectrometry, specific lipids, including cannabinoids, gangliosides and several other types, were detected in adult ganglia following mechanical damage to connected nerves. These observations motivated a search for possible effects of cannabinoids on neurite outgrowth. Exposing nervous tissues to Transient Receptor Potential Vanilloid (TRPV) receptor agonists resulted in enhanced neurite outgrowth from a cut nerve, while exposure to antagonists blocked such outgrowth.

**Conclusion:**

The experiments on the regenerating adult leech CNS reported here provide direct evidence of increased titers of proteins that are thought to play important roles in early stages of neural development. Our data further suggest that endocannabinoids also play key roles in CNS regeneration, mediated through the activation of leech TRPVs, as a thorough search of leech genome databases failed to reveal any leech orthologs of the mammalian cannabinoid receptors but revealed putative TRPVs. In sum, our observations identify a number of lipids and proteins that may contribute to different aspects of the complex phenomenon of leech nerve regeneration, establishing an important base for future functional assays.

## Introduction

A notable property of medicinal leech central neurons is their capacity to regenerate neurites and restore appropriate synaptic connections in the adult central nervous system (CNS): neurites that have been damaged or severed can sprout, establish *de novo* growth cones, extend long distances and reconnect specifically with normal targets [1]. In some instances, this process is greatly facilitated by the fusion of the proximal and distal regions of a cut axon [2]. A possible explanation for this useful attribute is that there is, in the adult leech, a continued presence and capacity for up-regulation of embryonic factors employed in early neuronal growth and maturation. Leech central neurons continue to expand their central and peripheral arbors throughout most of the life of the animal (animals get bigger following individual feedings), suggesting the possibility that the machinery for growth and addition of synaptic coupling may never be turned down or off completely in this invertebrate group. Alternatively, medicinal leeches may possess an unusual ability to enhance expression or repression of critical factors in response to signals produced internally by the damaged neuron or released extracellularly by the damaged tissues. These ideas can be tested by the detailed molecular analysis of changes in gene expression provoked by physical damage to the CNS, which is the subject of this report.

Over the past decade, Blackshaw and collaborators have implemented a differential screening strategy in order to assay directly for changes in gene expression at the transcriptional level that accompany neuronal regeneration in the leech [3]–[6]. Their approach is based on the use of subtractive probes, constructed by hybridizing cDNAs from regenerating and non-regenerating central ganglia and selecting these sequences enriched either in the regenerating sample (up-regulated genes) or in the non-regenerating sample (down-regulated genes). These probes were then used to screen cDNA libraries constructed from whole leech CNS or from identified micro dissected neurons [3], [6]. Thus far, this procedure has yielded a number of interesting results. For example, among sequences found to be up-regulated 24 hours following axotomy are the leech homologues of mammalian genes with established functions, such as genes encoding the cytoskeletal proteins actin, tubulin and Protein 4.1; thioredoxin (TRX), Rough Endoplasmic Reticulum Protein 1 (RER-1) and ATP synthase; and the neuron-specific protein synapsin. Others, such as the Cysteine Rich Intestinal Protein (CRIP), have been previously shown to be expressed in developing mammalian intestinal cells but not in adult regenerating nerve cells [3]. Other genes regulated by injury in the leech have counterparts in the mammalian genome but are not known to participate in mammalian regeneration processes. Two identified regulated genes, myohemerythrin [7] and the novel protein ReN3, are exclusively expressed in invertebrates. Still other regulated genes have no known homologues in vertebrate genomes, and these invertebrate-specific sequences are interesting in view of the different capacity for CNS repair in invertebrates such as the leech [3]. In order to investigate the key role of such genes in regeneration, siRNA studies have to be undertaken. RNAi studies at the single cell level have demonstrated the feasibility of such studies in leeches [8]. While these studies are at an early stage, it is quite clear that further analyses, particularly those examining the time course of injury-evoked changes in gene expression, have the potential to yield useful information, at a systems level, on the genetic programs underlying nervous system repair.

Studies of the modulation of gene expression at the mRNA level are clearly important, but they need to be complemented by observations of changes in protein levels and protein modifications using proteomics tools, as some of the critical effects of injury may be manifested in the regulation of translation and post-translational processing. Interestingly, several molecules detected in the differential transcription studies mentioned above have also been identified in recent proteomic studies of excised leech CNS challenged with bacterial toxins. These include cytoskeletal and metabolic proteins, foldases, calcium sensors, kinases and neurohemerythrin [9]. While this is a comparison of two levels of analysis - transcriptomic and proteomic - and responses to two different types of stress - mechanical trauma and septic shock - it is nonetheless satisfying that there is a convergence of results. An additional level of analysis needs to be considered, however. Lipids may also be critically involved in aspects of regeneration and repair, and these must be explored using lipidomic techniques.

In the work reported here, lipidomic and peptidomic assays coupled to molecular mass spectrometry imaging were used to explore molecular changes, by location and as a function of time after injury, that occurred in response to mechanical trauma and isolation of the leech CNS. A parallel embryonic study assessed whether molecules thus identified were also detected during development. The collected data provide an initial basis for the further exploration of a mechanistic understanding of the physiological processes involved in neural regeneration, including regulation of nitric oxide (NO) production, modulation of the innate immune response, the expression of neurotrophic and neurogenic factors, in the leech and other animals.

## Results

### Modulation of protein and peptide distributions during neural regeneration

#### Distributions of peptides/proteins in lesioned ganglion highlighted by MALDI imaging mass spectrometry

**Figure 1:**
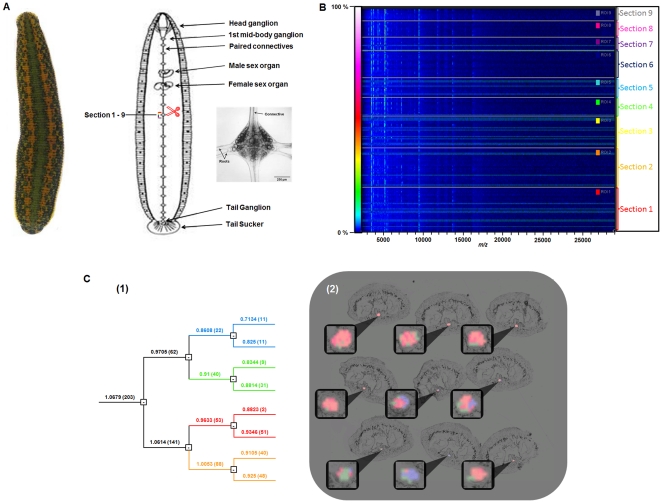
MALDI-MSI analysis of peptides in sections of regenerating adult CNS. **A**. Image of the dorsal aspect of a live adult specimen of a medicinal leech (*Hirudo verbana)*, head up (left part). Drawing features the ventral nerve cord, from the head ganglion to the tail ganglion, including the 21 midbody ganglia (right part). The location of the connective nerve crush, anterior to midbody ganglion 9 (red scissors), and the nine cross-sections (panel C2) are indicated. Example of a live midbody ganglion in culture (insert on the right). The interganglionic connective nerves and the nerve roots are labeled [19]. **B**. Two-dimensional representation of all the mass spectra (range *m/z*  = 1,000 to 30,000) corresponding to locations within a ganglion in the 9 sections (panel C2) shows variations in protein expression. The spectra are displayed as adjacent parallel lines in bands corresponding to each section (right of the graph). The number of pixels varies among sections, leading to bands of different widths. The spectra are normalized and ionic signal intensity is coded according to the color scale bar (0%: black to 100%: white) (left of the graph). (Section distribution for individual *m/z* values is diagrammed in Figure S4). **C**. (**1**) Full dendrogram shows the results of hierarchical clustering following principal component analysis of the MALDI-MSI dataset from 9 sections of the regenerating adult ganglion (panel B). The numbers in brackets correspond to the number of spectra per branch, and the horizontal numbers to the branch distances. (**2**) Reconstruction of selected dendrogram branches and corresponding images superposed on the 9 tissue sections, with each pixel color coded according to dendrogram branch. Only those pixels in the area of the ganglion are shown, superposed on the tissue image. Top row, sections 1-3, middle row, sections 4-6, bottom row, sections 7-9. Note that the number of pixels varies among sections.

**Figure 2:**
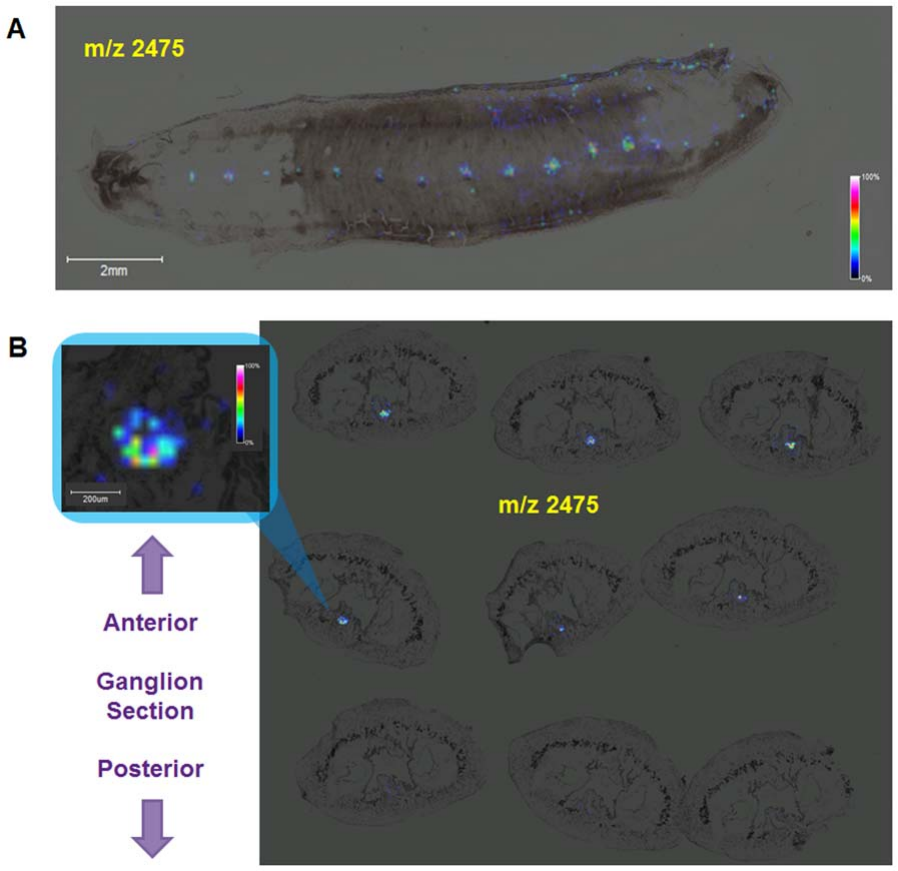
Expression of the ion at *m/z* 2475 in both embryonic and regenerating adult CNS segmental ganglia. **A**. Distribution of the *m/z* 2475 ion in a 12-day old leech embryo determined by MALDI-MSI of a dorsally-opened, whole mounted specimen. The ion is found at the highest abundance in the segmental ganglia of the ventral nerve cord. Head on the left, tail on the right, dorsal midline on the upper and lower margins of the dissected embryo. **B**. Distribution of the *m/z* 2475 ion in sections of the regenerating adult ganglion analyzed in Figure 1. The insert shows a magnified image of the data for section 4, with the abundance of the ion color coded according to the color bar at right. The peak corresponding to this ion is absent in a control adult (Figure S1), indicating a strong up-regulation of expression following injury.

In order to obtain a global map of peptides/proteins that might be involved in leech adult CNS regeneration at the ganglionic level, we performed MALDI mass spectrometry imaging (MSI) of sections of regenerating adult CNS following mechanical damage. Adult experimental animals received a crush in the connective nerves near the anterior margin of midbody ganglion 9, leaving the rest of the nerves between ganglia intact (**Figure 1A**). After allowing 6 hours for regeneration to be established, frozen 10 µm cross sections of whole animals were cut from anterior of the crush site to posterior of ganglion 9. Nine sections covering ganglion 9 from anterior (section 1) to posterior (section 9) were then imaged with MALDI-TOF in the region of the nervous system (**Figure 1C** **, panel (2)**). The spectra were taken in the range of *m/z* from 1,000 to 30,000 Da and normalized. To facilitate the comparison and interpretation of the data, the spectra for all sections were plotted together in a two-dimensional representation, with the spectra displayed as parallel horizontal lines, *m/z* values along the abcissa and intensity at each point represented by color (blue = low, white = high; scale on the left), and the set of spectra for each section separated from each other by thick lines (**Figure 1B**). The spectra obtained for all 9 cross-sections of the ganglion were then subjected to principal component analysis (PCA) followed by hierarchical clustering. The dendrogram of the clustering results shows that spectra represented by some branches of the dendrogram correspond to more anterior locations while others appear to be more posterior and are more numerous (**Figure 1C** **and Figure S4**). These statistical analyses highlight two distinct areas, corresponding to the anterior (red, orange) and the posterior (blue) parts of the ganglion, and show significant differences in terms of their nature and level expression. This suggests that peptides are produced by neurons that reside closer to the lesion or that factors are differentially transported towards the damaged area. For example, the peptide with the *m/z* of 2475, previously detected in the embryo nerve cord (**Figure 2A**) is also present in the adult regenerating ganglion with an anterior expression bias (**Figure 2B**), whereas it is absent in controls (non regenerating adult CNS segmental ganglia, **Figure S1**). This peptide, which has been recently identified as a fragment of a novel intermediate filament protein, *Hm*IF4 [10] and has the N-terminal sequence GTRTMERSVRTSSQYASGGPMPN, provides evidence for the idea that embryonic factors are re-expressed or up-regulated during the process of regeneration.

**Figure 3:**
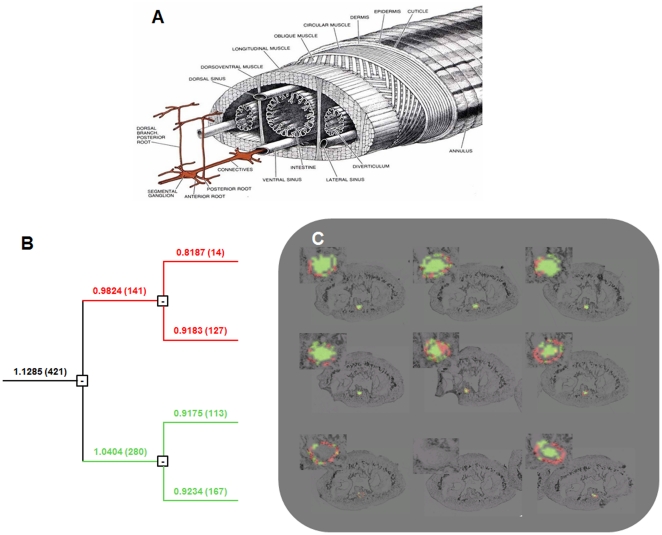
Hierarchical clustering of spectra from 9 sections of a regenerating ganglion and surrounding blood sinus. **A**. Drawing of the leech structure features the ventral blood sinus surrounding the nerve cord [89]. **B**. Full dendrogram of all spectra in the ganglion dataset yields two main branches, colored red and green, that segregate into different domains in the images (panel C). **C**. Reconstruction of selected dendrogram branches and corresponding images shows that the upper branch (red) corresponds mainly with the blood cells (annulus around the central region) while the lower branch (green) corresponds mainly to cells in the CNS region. In some sections, however, cells characterized by the blood sinus peptide profile (red) appear to have migrated into the area of the ganglion (sections 5 and 6 in particular).

Since the leech nerve cord resides within the ventral blood sinus, it is important to assay whether factors produced at the lesion site might be derived from blood cells instead of the CNS tissues proper. In this context, MSI was performed on the sections of an experimental adult, but this time including in the analysis both the tissue of ganglion 9 and the surrounding area of the blood sinus. PCA followed by hierarchical clustering yield a dendrogram with two major branching domains (red and green branches; **Figure 3A**). When these are related to the corresponding tissue locations, one set of branches (green) clearly corresponds to ganglionic cells while the other (red) is present in the cells within the surrounding blood sinus (**Figure 3B**). The distributions of these two proteic profiles in some of the sections (sections 5 and 6, **Figure 3B**) suggest that cells contained within the sinus also participate in the regeneration process by migrating into the ganglion. Observation of two distinct profiles, specifically highlighted in the blood sinus or in the ganglion in control sections of adult leech, seems to confirm this hypothesis (**Figure S2**). These observations support the concept that several classes of cells participate in the regeneration processes including neurons, microglia and blood cells. In the course of regeneration, communication among these cells might involve different classes of molecules at each step of this biological process. We previously demonstrated that, early in the process, regenerating neurons produce chemoattractive factors related to chemokines, *e.g., Hm*EMAPII [11], *Hm*IL16 [12] and *Hm*C1q [13], which are present in both leech and human microglia [11]–[13] and antimicrobial-neurotrophic factors, *e.g.*, neuromacin and *Hm*-lumbricin [14]. Finally we recently characterized these blood cells as hemocytes able to produce neurotrophic factors related to the antimicrobial peptides [15], including *Hm*-lumbricin, *Hm*-theromyzin and *Hm*-theromacin [16]. The sum of these observations argues for a critical role of blood cells in neuronal regeneration.

#### Peptide/protein identification

Two strategies were employed to assay changes in the expression of peptides and proteins resulting from mechanical damage to the CNS: a global approach, using a “bottom-up” application to observe the adult CNS in the course of regeneration but focused on the peptides or proteins identified specifically during neurogenesis, and a targeted approach, based on Differential-Display HPLC (DD-HPLC), neurite outgrowth and antimicrobial tests. The main goal of these two strategies was to identify candidate peptides/proteins which are likely to have important functions in the process of adult CNS regeneration.

The Bottom-up strategy was based on the hypothesis that many factors that are important and useful in the reconstruction of the nervous system after injury ought to also be present during the initial “construction” phase, i.e., during neural differentiation. Thus, the criteria for selecting specific peptides or protein fragments for further analysis in this set of experiments was detectability during neurogenesis as well as during regeneration. For this purpose, we extracted peptides from stage E12 embryos (a midstage in neurogenesis) and from adult nerve cords 6h after lesioning the connective nerves *in vivo*. Following acidic extraction and sep-pack prepurification, the 50% AcN fractions were subjected to trypsin digestion before being separated in nanoLC and characterized in ion-trap using MS/MS mode, and analyzed using InsPecT software [17] against predicted translated sequences in the *Hirudo medicinalis* EST database [18] and *Helobdella robusta* genome (http://genome.jgi-psf.org/Helro1/Helro1.home.html). Each identified peptide sequence was then blasted using NCBI p-Blast against Hirudinidae species. **Table 1** presents a sample of the identified proteins present in both adult nerve cords in regeneration (absent in control) and leech embryos (sequence alignment **Figure S3**) based on identified trypsin sequenced peptides after nanoLC separation. These can be grouped into six different functional categories: antimicrobial-neurotrophic factors, chemoattactractant factors, axonal guidance factors, gap junctional proteins, homeobox gene factors, and Ig superfamily proteins, representing the broad functional requirements of these dynamic processes.

**Table 1:** Proteins detected in both regenerating adult nerve cord and in embryos.

*Identified Proteins*	*Identified Sequences*
**Destabilase I**	LIAQLKVNSQFTDSCLR
**Neuromacin**	GLFMYMIGFLLLSDCNSNWATTRLDKSSLIGILWKNCNER
**C1q domain**	DRIFSSGSSGEDDDDDDDDDSKNSNYLLTKLGPRGTAGPDGVVGQKGDSGELGPPGLPVTTSTRNMLEGDDR
**Interleukin 16 related Protein**	RSEPRQNMHLRRKD.FTKPRKNCLASMTS.RS.SFFAGALGVTFCVEGGR
**P43/EMAPII**	FIDIQGFLCPLRWTGGFAVSKTQLLGGTPRHSKALQFCRE.AVDGVQIRPPAGVSVPGRGAADPRAVVAGGLSLCPQGADQGRNPSSKQQSSAVCSTVVCK
**Filamin**	ESGSGIPTTATEAVDGVQIRKMRSKSVPSTGESFTSTSTGTPGMETFTSSAPKMGDQGGITKNTPCTGSFTRWTGGFAVSKTQLLGGTPRHSKALQFCRSADRECPDFLTTKAFGTSPESGSGIPTTATKEAVDGVQIRPPAGVSVPGRGAADPRAVVAGGLSLCPQGADQGRQGEDAGKFAATEIKVTAVDASSGQHVDSSIDKFSEFATQISIQTIPFIASLLLLCTMAGDLALVIRLSVSCSKRSLPGLGSGSSPLVGRGNGRGGRNPSSKQQSSAVCSTVVCKPDLDAISGELVK
**Hillarin**	A.SLLICNMNSLLSDCSLLERNLVGDKGRNLKI.LAFFLFQKIFKP.ASEALACSSSSLVIAMTLPGKKLSSGRSSRASCSSSSNSAKRSSSSTSPNPTGFEIAFEISKI.LAFFLFQKIFKYNLQIILL.YR
**Macrolin**	VHQEQNLMLISKNSNYLLTKGAVAAAVCTLNSGFLVAEENTRDDGLGHLGVHGRIVGRIDGTGAECGRIGNSLRPASIHSSDLEARSWGLNQRLVTGLISYSADLKDGQVTTSTRNMLEGDDRGTTEYPFK
**Tractin**	SLRVEDVIKEGNNPLQQIHRCGKGLPLLIFLPGGGPTLPTKNFKIRDLLVSSSATSVVGWPEIRF.LLLIATDDSLQLESLHDAPNKLNNNYKNIQKA.ASVSSLASKKNIFVNTFHVKG.NRHSPGQEVQGGRGSLRVEDVIKEGNNPLQQRIHRCGKGLPLLIFLPGPIDMEGGGGGGPTL.R
**Innexin 4**	ILKLLYKETNTQFSGSLVSVARQNEMNRYSMGDQGGITK
**LOX2**	PRQNMHLRRKADFSLYQRINISSHFDFTKPRKNCLASMTSR
**LAR2 Ig domain**	VHHRGPSCGSDRPSGDCTRWYPRSPHTNSRIVNSVNSRNGSVSGHSVRAKHDKLMGSGDQGKSIAMVESFRGQGPEGGLPSDPAPDVRAGRTKCNFQVFQLLLNGIGALNSRW.QSLRKDSKSSLLVNAGSASQGKVNVAMR
**Leech Ena**	DKAKQKGEDFENANCKLCWGSSACDVLPSLPAHSLAALTSPPPTPSFK

MS/MS analyses were performed by nanoLC/Trap after trypsin digestion. Identifications were performed by searching the *Hirudo medicinalis* EST databank and *Helobdella robusta* genome and screening using INSPECT software.

**Figure 4:**
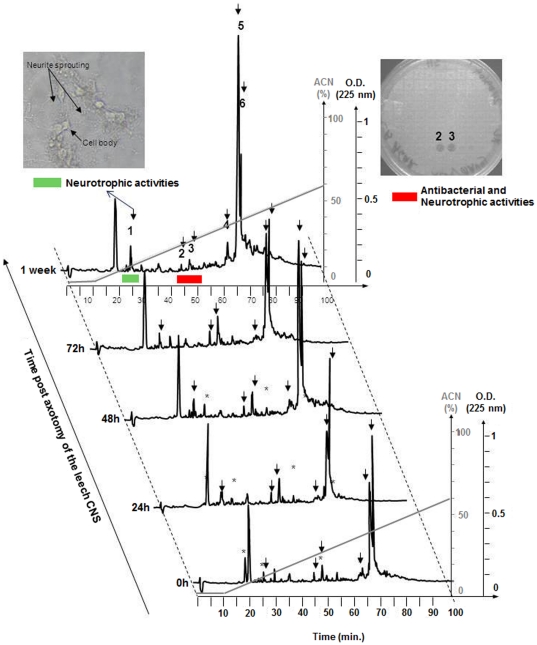
DD-RP-HPLC of leech axotomized nerve cords acidic extracts at different times after connective nerve trans-section. After prepurification by solid phase extraction, the 60% AcN eluted material is loaded onto a C18 column. Elution is performed with a linear gradient of AcN (dotted line) and absorbance was monitored at 225 nm. Each individually collected peak is tested for its neurites outgrowth (example in upper left insert) and antimicrobial (example upper right insert) activities. The peaks that showed temporal variation in intensity, whether they produced antimicrobial or neurotrophic activity or not, are further purified and the peptides are identified by MALDI-TOF mass spectrometry and Edman degradation. Sequence results and peptide identification are shown in Table 2.

Differential Display (DD)-HPLC analyses coupled to neurite outgrowth and antibacterial assays were performed on peptidic extracts of nerve cords maintained in culture for different times post axotomy (**Figure 4**). Comparison of the HPLC spectra revealed six peaks in the range considered that changed significantly in the course of the experiment, from T0 to 1 week post axotomy. The identification of the peptides contained in these fractions was performed by combining further HPLC purification, mass spectrometry analysis and Edman degradation. The *m/z* values, amino acid sequences and proposed identities are presented in **Table 2**. The first peak (Peak 1; **Figure 4**), which increases in size with time post-axotomy, was also found to exert neurotrophic activity in leech neurite outgrowth tests (**Figure 4**, left upper insert). It represents a value *m/z* 3974 and has the following N-terminal sequence, obtained by Edman degradation: RRLPKFPKFPKFPKLPDWPQWPEWPQE, Querying the *Hirudo* Genome, the complete sequence is the following:RRLPKFPKFPKFPKLPDWPQWPEWPQEQQLQEWPPEEGLSE. This is a novel peptide and shows antibacterial activity in our assay (see Methods) which we have named *Hm*-ABP3. Two other peaks selected for further analysis also turned out to be antimicrobial peptides (AMPs), but two that we had previously identified in the leech CNS, *Hm*-lumbricin (Peak 2, **Figure 4**) and *Hm-*neuromacin (Peak 3, **Figure 4**) [14]. Both of them display antimicrobial activity (upper right insert, **Figure 4**), and both show neurotrophic properties in neurite outgrowth tests as we have previously shown [14]. The remaining three peaks also correspond to previously identified leech proteins. Peak 4 (**Figure 4**) represents a peptide sequence related to the intermediate filament gliarin [9], while Peaks 5 and 6 are both related to neurohemerythrin [7]. Neither antimicrobial nor neurotrophic activities were detected for any of these three molecules (data not shown).

**Table 2 pone-0018359-t002:** Peptide sequences obtained after DD-HPLC, tested in neurite outgrowth or antimicrobial tests and sequenced by automated Edman degradation.

*Peak #*	*m/z*	*Sequences*	*Biological Activities*	*Blastp*
**1**	3974	RRLPKFPKFPKFPKLPDWPQWPEWPQES	Neurotrophic	Hm-ABP3
**2**	6709	DXYEDWSRXTPGTSFLTGILXKDXSR	Antimicrobial	Neuromacin
**3**	6443	FSKYERQKDKRSYGERFS	Antimicrobial	Hm Lumbricin
**4**	11342	QXLGMSSSTIRREKTIQYGNAY		Intermediate filament gliarin
**5**	13721	GFEIPEPYVRDEDFGVFYFNLDELH		Neurohemerythrin isoform
**6**	13814	GFEIPEPYVWDESFKVFYENLDEXHKGLFQAIFN		Neurohemerythrin

In contrast to the kinetics of *Hm*-ABP3, the levels of *Hm*-lumbricin and *Hm*-neuromacin decreased a week post axotomy, after increasing for at least 3 days. This observation, in addition to their constitutive expression in the nerve cord, suggests that these two AMPs are mainly involved in the early events of neural repair. Similar to *Hm*-ABP3, the gliarin fragment and the neurohemerythrin isoforms appeared to become more abundant throughout the period examined, suggesting that their roles might become more important as the process of regeneration is established, but might not be as significant immediately after the lesion is made, when the neuroinflammatory response occurs [11]–[14], [19], [20]. Curiously, the two neurohemerytrin isoforms are differentially regulated, possibly reflecting different functions [7], [9]. These results are consistent with the data obtained by the bottom-up approach, supporting the tentative conclusion that gliarin and antimicrobial-neurotrophic factors are implicated in the adult nerve cord regeneration. In addition, our observations complement those obtained by genomic and 2D-Gel proteomic studies [3], [5], [7], [9] (**Table 3**). By comparison, Blackshaw and collaborators have reported, in studies using subtractive cDNAs library from regenerating and non-regenerating central ganglia, the presence of leech homologues of mammalian genes with established functions, including the cytoskeletal proteins actin, tubulin and Protein 4.1, ATP synthase, the neuron-specific protein synapsin, Cysteine Rich Intestinal Protein (CRIP), myohemerythrin and a novel protein ReN3, exclusively expressed in invertebrates [3], [4], [21], [22]. To investigate the possible roles of such genes in regeneration, siRNA studies have to be undertaken [6]. Similarly on-going proteomic studies of the leech CNS performed by our group have shown that some molecules detected by transcriptomic approaches, *e.g.* cytoskeletal and metabolic proteins, foldases, calcium sensors, kinases and neurohemerythrin (reflecting specific cytoskeletal rearrangements linked to cell migration), vesicular trafficking as well as the modulation of synaptic activity, are also observed in excised adult ganglia challenged by bacterial toxins [7], [9].

**Table 3:** Leech nerve cord proteins implicated in adult neuroregeneration using Proteomic (Bottom-up, DD-HPLC, 2D-Gel analysis) and Transcriptomic approaches.

*Protein Categories*		*Bottom-up Strategy*	*2D Gel Proteomic Strategy * [*9*]	*DD-HPLC Strategy*	*Soustractive cDNA library * [*3*]
**Cytoskeleton**	Microfilaments		Tropomyosin	Gliarin	Protein 4.1
	Intermediate filaments	FilaminGliarinMacrolin	Gliarin		ReN3Synapsin
	Microtubules				α &β tubulins
**Calcium**	Calcium sensor		NCS2Neurocalcineurin		Calmodulin-like
	Others				
**Metabolism**	AA/nt metabolism		AA deshydrogenase		ATPase inhibitor
	Energy		ATP synthase β unitAcetyl tranferase		
**Hsp** **&** **Chaperones**	Cyclophilin/PPIPDI		CyclophilinPDI		
	Others				HSP 90
**Metal** **oxydation**	Resp. molecules		NeurohemerythrinCOX I		MyohemerythrinCOX I
	Others				
**Immune effectors**	Cytokines	Il-16C1qEMAP-II			CRIP
	PAMs	Neuromacin		Hm-lumbricinNeuromacinCathelicidin-like	
**Axonal guidance**	Ig-superfamily	TractinHillarin			
	Chemotrophic factors	NetrinLena			
	Tyrosine phosphatase receptor	LAR2 ectodomain			
**Morphogenesis**	Homeobox gene	LOX2			
	Gap junction	Innexin 4			

(AA, aminoacyl; CRIP, Cysteine Rich Intestinal Protein; COX I, Cyclooxygenase I; nt, neurotransmitters; PPI, peptidyl-prolyl cis-trans isomerase).

#### Time course changes of proteome of lesioned ganglion

Based on previous proteomic [7], [9] and genomic studies [3], [21] and peptides quantifications obtained by DD-HPLC or from the identification of peptides in trypsin digests by MS/MS after nanoLC separation (see Methods), it is possible to propose a possible scheme for how the factors implicated in the regeneration processes might be involved from lesioning (time 0) to day 7. As presented in **Table 4**, immune factors implicated in neuroinflammation, such as antimicrobial peptides and chemoattractants, are released early (between 1 h to 6 h) in order to activate microglial cells and initiate the regeneration process. Inflammation is necessary in order to start the wound healing at the periphery. In the brain, neuroinflammation is also necessary for activating specific cells and for the release of regeneration messengers. Thus, the detection of EMPAII [11], IL16 [12], C1q [13], [20] and the antimicrobial peptides [14] is expected. The second wave of molecules seems to be related to physical reconstruction and tissue repairing, with the up-regulation of cytoskeleton proteins and modulatory components like myohemerytin, filarin, gliarin, macrolin, leech CAM, and tractin [7], [23]–[27]. All of these proteins support the neuronal outgrowth that will be responsible for reconnecting neurons. Then neuronal sprouting and axon guidances proteins, like liprin, hillarin, leech Ena, netrin and syntaxin [23], [28]–[30] can be detected. This is then followed by a phase in which, during the course of tissue repair, signaling pathways are reestablished through the heightened expression of novel hemi-channels related to gap junctions formation (innexins), synapsin, ReN3, voltage-gated Na channels isoform [3], [21], [31], [32].

**Table 4:** Protein identification in time course of regeneration processes based on complementary techniques (2D-Gel [7], [9], DD-HPLC, Bottom-up proteomic and soustractive DNA libraries [3], [21]).

Class of Proteins	Protein Name	0 h	1 h	6 h	24 h	48 h
Immune Factors						
	IL-16, EMAP II	X	X	X		
	Antimicrobial	X	X	X	X	
	C1q	X	X	X		
	CRIP				X	
Cytoskeleton						
*Intermediate Fil.*	Filamin			X	X	X
	Gliarin		X	X	X	X
	Macrolin			X	X	X
	Leech CAM			X	X	X
	Tractin			X	X	X
	Protein 4.1.				X	
	Synapsin			X	X	
	ReN3				X	
*Microtubules*	Tubulins				X	
Axon Guidance						
*Ig Superfamily*	Hillarin			X	X	
	Leech ENA			X	X	
	Liprin			X	X	
*Chemotrophic F.*	Netrin			X	X	X
	Syntaxin			X	X	X
	Destabilase		X	X	X	X
*TRP*	LAR2 e.d			X	X	X
HSP&Chaperones						
	Cyclophilin			X		
	PDI				X	
	PPI				X	
	HSP90				X	
Metal oxydation						
	Neurohemerythin		X	X	X	X
	COXI		X		X	
Metabolism						
*AA/metabolism*	AA Deshydrogenase			X	X	
	ATPase Inhibitor				X	
*Energy*	ATP Synthase			X	X	
Morphogenesis						
*Homeobox gene*	LOX2			X	X	X
*Gap Junction*	Innexins			X	X	X
Calcium						
*Calcium Sensor*	NCS2		X			
	Neurocalcineurin			X		
*Others*	Calmodulin-like				X	

### Changes in lipid expression following mechanical trauma

**Figure 5:**
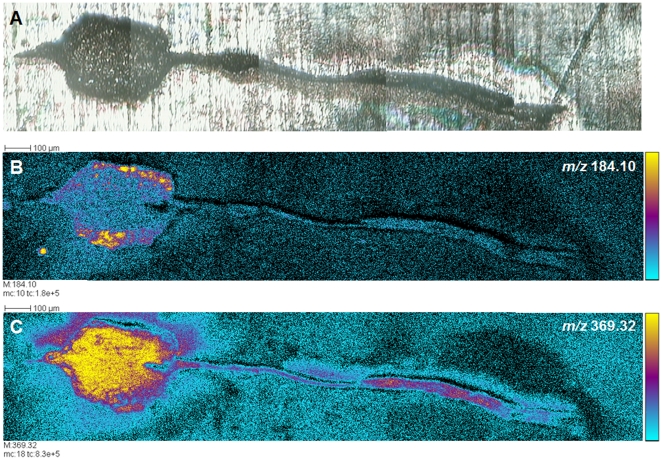
ToF-SIMS analysis of lipids in a portion of control leech nerve cord, including a ganglion. **A**. Optical, transmitted light low-resolution image of unstained tissue (ganglion and parts of attached nerve) prior to ToF-SIMS analyses. The ganglion is placed in gelatin before being sectioned at 10 µm. The slices are deposited onto silicium target. **B, C**. Tissue distributions of two ions, *m/z* 184.1 and *m/z* 369.32, corresponding to the phosphocholine ion and to the cholesterol fragment ion [M+H-H_2_O]^+^, respectively. The phosphocholine ion can be observed mainly in the outer areas of the ganglion, where neuronal somata and glial packets are located, while the cholesterol fragment ion is also found throughout the central neuropil. Five adjacent individual images of 500×500 µm^2^ where assembled end-to-end to create these images. Color scale bars, with amplitude in number of counts, are indicated to the right of each ion image. The amplitude of the color scale corresponds to the maximum number of counts *mc* and could be read as [0, *mc*]. *tc* is the total number of counts recorded for the specified *m/z* (sum of counts in all the pixels).

Beside lipids are also known to be involved in inflammation modulation and brain regeneration [33]. As expected, screening of the leech EST database revealed the presence of partial transcripts for many enzymes involved in lipid metabolism, including phospholipase A2 (cytosolic and calcium-independent forms), phospholipases D3 and C, lysophospholipase, and arachidonate 15-lipoxygenase type II (**Table 5**). The presence in the leech nervous system of some of the major lipid enzymes implicated in brain regeneration [34], [35] led us to undertake a thorough analysis of lipids present in the leech nerve cord during the course of regeneration using molecular imaging by ToF-SIMS technology (**Figures 5** **and** **6**). Besides ToF-SIMS imaging, a targeted strategy was undertaken using MALDI-TOF to explore the distribution of endocannabinoids (**Figure 7**) because of their well-known involvement in mammalian brain regeneration through vanilloid receptor activation [36]–[39].

**Figure 6:**
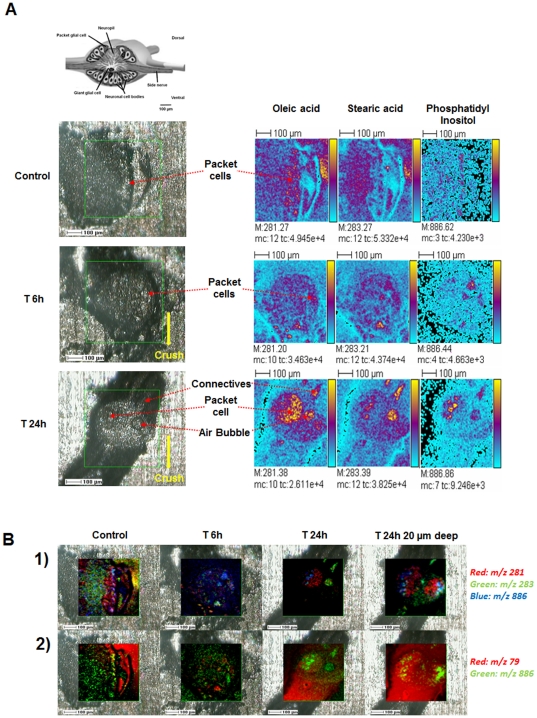
ToF-SIMS lipid ion images of leech ganglia in course of regeneration. **A**. Drawing of the structure of ganglion (upper left of the panel) [19]. Optical, transmitted light low-resolution images of control, 6 h and 24 h regeneration ganglia included into gelatin 10% before sectioning (lower left of the panel). Highlighting the changing distributions of oleic acid carboxylate (*m/z* 281.2), stearic acid carboxylate (*m/z* 283.2) and phosphatidylinositol PI34:2 (*m/z* 885.5) in these ganglia in course of regeneration by ToF-SIMS imaging (right of the panel). Color scale bars, with amplitude in number of counts, are indicated on the right margin of each ion image. The amplitude of the color scale corresponds to the maximum number of counts *mc* and could be read as [0, *mc*]. *tc* is the total number of counts recorded for the specified *m/z* (sum of counts in all the pixels). **B**. Lipid ion images (color overlays) of leech ganglion in course of regeneration (0:control, 6 h and 24 h regeneration). (**1**) Oleic acid carboxylate (*m/z* 281.2), stearic acid (*m/z* 283.2) and phosphatidylinositol PI34:2 (*m/z* 885.5) and (**2**) Phosphatidylinositol (*m/z* 885.5) and phostate (*m/z* 78.9).

**Table 5:** Leech Brain phospholipases and lipoxygenases obtained from *Hirudo medicinalis* nerve cord EST library (http://www.cns.fr/spip/Hirudo-medicinalis-collection-d.html).

*Contig Number*	*Protein*	*Homology*	*e Value*
EN-133k-group2267.Contig1	Phospholipase D3 (EC 3.1.4.4) (PLD 3) (Choline phosphatase 3)	*Bos taurus*	1e^−100^
EN-133k-group1648.Contig4	Phospholipase A2, group IVB (Cytosolic)	*Xenopus tropicalis*	1.3 e^−74^
EN-133k-group574.Contig2	calcium-independent phospholipase A2(iPLA2)	*Mus musculus*	1.4 e^−59^
EN-133k-group179.N_gs_28394	Lysophospholipase	*Argas monolakensis*	6.2e^−59^
EN-133k-group11049.Contig1	Phospholipase C	*Aplysia californica*	9.7e^−51^
EN-133k-group12326.N_gs_44143	Phospholipase D	*Aedes aegypti*	2.5e^−48^
EN-133k-group501.Contig1	Arachidonate 15-lipoxygenase type II	*Mus musculus*	9e^−47^

#### Distributions of lipids in lesioned ganglia highlighted by ToF-SIMS imaging

ToF-SIMS imaging of the leech nerve cord enables the detection of a great number of lipids, including lipids implicated in regeneration [40]. To explore how these distributions change over time, we imaged lipids in the nerve cord near the site of the lesion at different times after trauma (T0, T6 h, T24 h) in order to assay changes that correlate with different stages in the process of repair. **Figure 6A** presents the distributions, in sections of adult CNS, of some examples of lipids modulated in the course of regeneration: oleic acid carboxylate, mono-unsaturated omega-9 fatty acid (*m/z* 281.2), stearic acid carboxylate (octadecanoic acid: *m/z* 283.2) and the PI38:4 phosphatidylinositol (*m/z* 886.5) [40]. At T0h (control, **Figure 6A**), the patterns of expression of oleic and stearic acids showed little overlap within the ganglion, but at T6 h, both lipids were weakly expressed in the packet cells. By T24 h, stearic acid showed stronger expression in the connective nerves and weaker within the ganglion in comparison with oleic acid, which appeared to accumulate in packet cells and be poorly expressed in the connective nerves. The phosphatidylinositol ion was also detected at lightlevels in the control ganglion at T0 h compared to the phosphate ion (PO_3_^−^, *m/z* 79), which was broadly distributed in nervous tissue (**Figure 6B**). After 6 hours of regeneration, phosphatidylinositol appeared in cells near the connective nerve that links the ganglion analysed to the ganglion anterior to the lesion site, whereas at 24 hours of regeneration it was exclusively localized in packet cells in the experimental ganglion.

#### Endocannabinoid involvement in nerve cord regeneration

**Figure 7:**
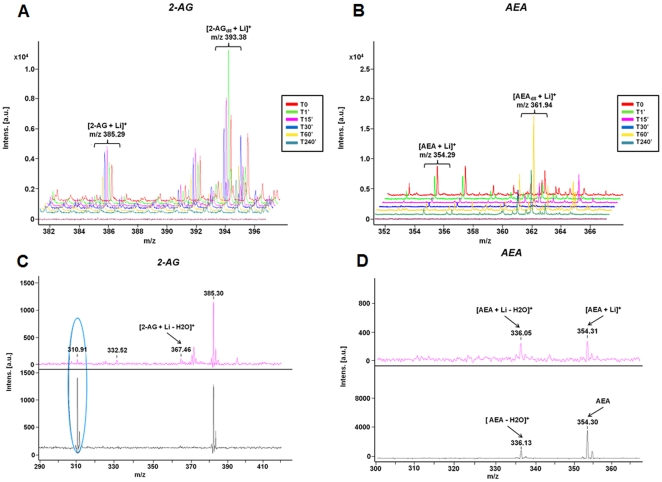
Levels of cannabinoids change as a function of time after injury. Time course MALDI-TOF measurement of cannabinoids in injured nerve cords from time 0 to time 240 minutes. **A**. Levels of 2-AG and 2-AG deuterated (D_2_O) with lithium chloride addition after the nerve cord lesions. **B**. Levels of AEA and AEA deuterated (D_2_0) with lithium chloride addition after nerve cord crush. **C**. MALDI-MS spectra of standards of 2-AG and 2-AG with lithium chloride addition. **D**. MALDI-MS spectra of standards of AEA and AEA with lithium chloride addition.

Along with these more global studies, we also investigated a specific family of lipids, the cannabinoids, which are known to be involved in neurogenesis as well [41]. We previously established in leeches the presence of a fatty acid amid hydrolase and monoglyceride lipase [42], and have shown some of their effects on inflammation regulation [37], [43], [44]. We also demonstrated the presence of the endocannabinoids anandamide (AEA, N-arachidonoylethanolamine, 21.5+/−0.7 pmol/g) and 2-arachidonoylglycerol (2-AG, 147.4+/−42.7 pmol/g), and of the biosynthetic precursor of anandamide, N-arachidonylphosphatidyl-ethanolamine (16.5+/−3.3 pmol/g), in the leech central nervous system (CNS). Anandamide-related molecules such as N-palmitoylethanolamine (32.4+/−1.6 pmol/g) and N-linolenoylethanolamine (5.8 pmol/g) were also detected. In this study, we used MALDI-TOF and lithium adducts (see Methods) to detect the release of AEA and 2-AG during regeneration. Analysis of temporal changes in MALDI mass spectra showed an increase for AEA and 2-AG signals, occurring within less than 15 minutes after the lesion (**Figure 7**), reflecting a very quick release of these lipids. Both AEA and 2-AG were present and peaked in the first hour (AEA at ∼1 hour, 2-AG within minutes) in response to nerve cord damage. This behavior is consistent with our previous demonstration that these endocannabinoids can modulate leech brain inflammation [38], [42]–[45] and can act as chemoattractants for microglial cells [43], [46].

**Figure 8:**
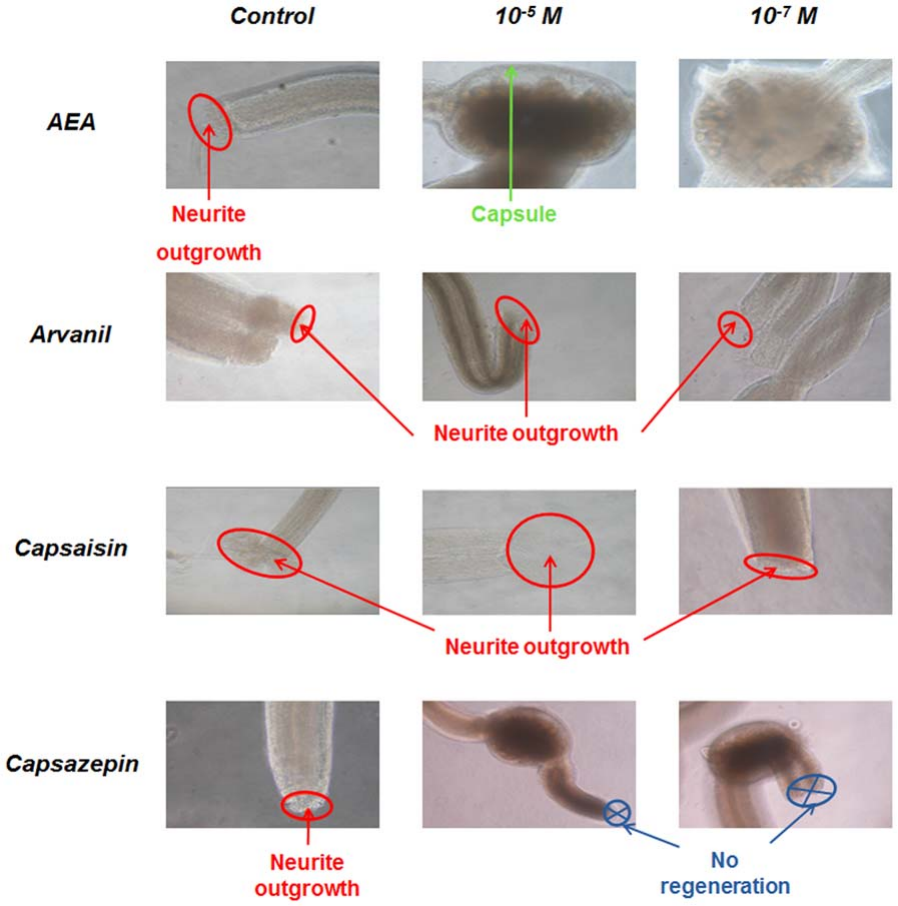
Cannabinoids affect neurite outgrowth from cut nerves in cultured segments of the CNS. *In vitro* leech nerve cord culture and neurite outgrowth assays with arvanil, capsaicin, capsazepin at concentration of 10^−5^M or 10^−7^M. Neurite outgrowth is indicated as well as the ones non regenerating.

To assay for possible physiological activity of these cannabinoids in regeneration, we conducted a series of *in vitro* studies with several cannabinoid and vanilloid receptor agonists and antagonists. Specifically, nerve cord segments were placed in culture, subjected to different concentrations of AEA, capsaicin, arvanil and capsazepin, and checked for neurite outgrowth from a cut connective nerve at various intervals after lesion (**Figure 8**). The results show neurites sprouting from the cut nerve in less than a week for the control preparations and in less than 3 days for preparations exposed to either of the agonists, capsaicin and arvanil, whereas no outgrowth was observed with exposure to either of the antagonists tested, AEA and capsazepin. For the latter molecules, a repair process has apparently occurred, since a brown coloration can be observed that indicates a wound healing process is taking place instead of neuronal regeneration.

#### Endocannabinoid effects are mediated by vanilloid but not cannabinoid receptors in the medicinal leech

To determine how the effects of release or exposure of endocannabinoids might be mediated in the regenerating leech CNS, we undertook a careful and thorough exploration of all available leech transcriptomic and genomic databases for orthologs of receptors known to bind these lipids. These included the publicly available *Hirudo sp.* transcriptome [18] and the *Helobdella* databases [47] as well as a *Hirudo verbana* genomic database currently being assembled in our laboratory (T. Gaasterland, private communication). This *Hirudo* genome database was constructed using 73 M paired-end reads from genomic DNA inserts of length 350 bases and 56 M single reads, with reads of length 100 bases, obtained from libraries prepared with DNA from two adult Hirudo verbena specimens and sequenced on an Illumina Genome Analyzer IIx instrument. The reads, containing a total of 12.9B unfiltered bases, were filtered and errors corrected with Euler-NR sequence preparation modules [48] and assembled with Velvet [49], [50] using parameters obtained through Velvet Optimizer [51]. The resulting 57,700 assembled contigs have an average read-depth coverage of 26x, contain 165 Mbp, and should include sequences of nearly all medicinal leech genes.

**Figure 9:**
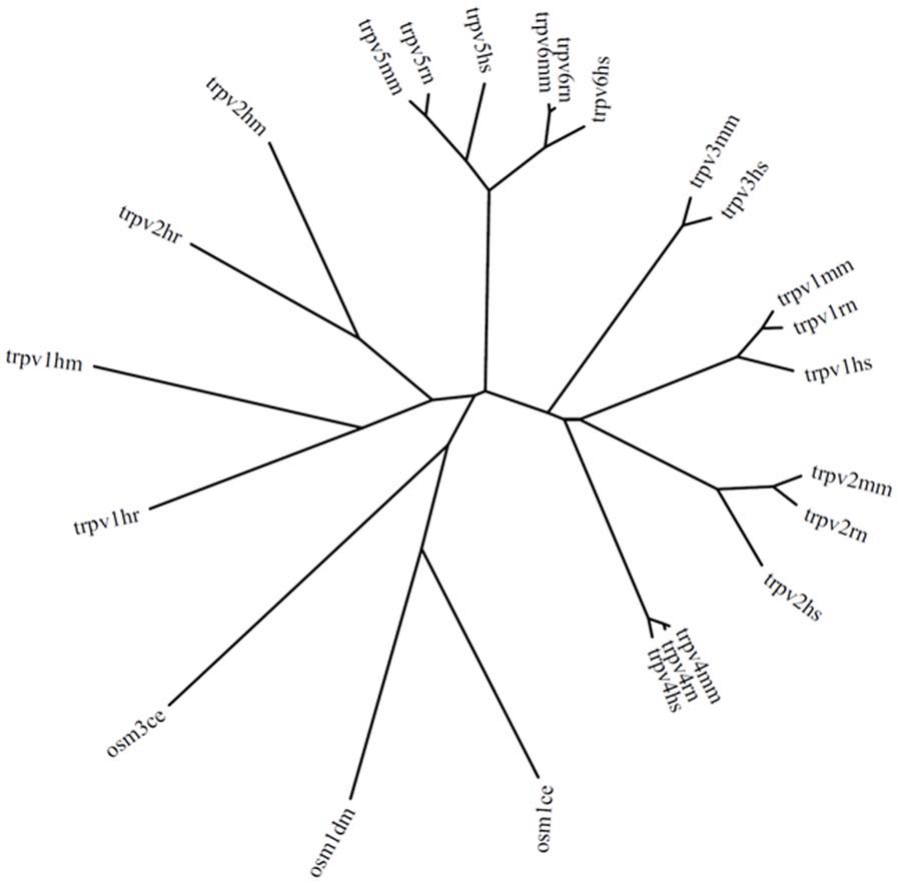
Phylogenetic tree showing *Hirudo*, *Helobdella*, mouse, rat, Human TRPVs and *C. elegans*, *D. melanogaster* OSMs. Leech TRPVs are slightly less diverged from mammalian TRPVs than those of *C. elegans* and drosophila and are closest to mammalian TRPV5 and TRPV6. (ce, *Caenorhabditis elegans*; dm, *Drosophila melanogaster*; hm, *Hirudo medicinalis*; hr, *Helobdella robusta*; hs, *Homo sapiens*; mm, *Mus musculus*; rn, *Rattus norvegicus*).

Curated protein sequences for cannabinoid receptor (CB1 and CB2) sequences available in GenBank were aligned against the genome contigs using Tblastn and failed to detect any leech orthologs, leading us to conclude they are not present in leeches, as has been reported in other invertebrates. Mammalian sequences from another group of receptors reported to bind endocannabinoids, the Transient Receptor Potential Vanilloid (TRPV) receptors, were then used as query sequences. Tblastn alignments [52] identified two distinct full length putative medicinal leech orthologs in the *Hirudo* genome, both of which also had orthologs in *Helobdella*. A third partial protein fragment with an ankarin repeat domain was identified in *Hirudo* and may represent a third *Hirudo* TRPV. Exons were extracted from contigs and concatenated to form *Hm*TRPV1-3 shown in **Figure S5**. A partial transcript for *HmTRPV1* was also found in the *Hirudo* transcriptome database [18]. An evolutionary comparison (**Figure 9**) shows that the *Hirudo* TRPVs are diverged from mammalian TRPVs, as are the related OSM1 and OSM3 proteins from *C. elegans* and *D. melanogaster*.

On the basis of these findings, we propose the hypothesis that CNS regeneration involves endocannabinoids acting through leech vanilloid receptors. This hypothesis is currently being tested and the results will be reported in a subsequent publication.

## Discussion

The medicinal leech is a very interesting model system for the study of the mechanisms of neural regeneration because of the capacity to regenerate the CNS throughout its life. The continued presence of embryonic factors necessary for neuronal growth and maturation and the ability to induce expression or repression of critical factors in response to signals released by damaged tissues are possible explanations for this useful property.

Another aspect of these animals is the continuous expansion of their central neurons. This suggests that the machinery for growth and addition of synaptic coupling may never be turned down or off completely in leeches, in contrast to mammals, in which not only are many embryonic growth-promoting molecules and their receptors apparently no longer present in the adult, but the adult CNS produces various growth-inhibiting molecules that are not present in the embryo or neonate [53]. Comparison of the mechanisms of regeneration in leeches and mammals should yield some insight into which components might be artificially modulated, and how, in order to enhance regeneration in the mammalian nervous system.

The observations reported and discussed here were conceived as a starting point for applying MALDI imaging to the discovery of the protein and lipid components involved in the process of neural regeneration in the medicinal leech model system. MALDI imaging analyses provided an initial overview of the presence, abundance and distribution of many peptides/proteins in the range *m/z* 1,000 to 30,000 in regenerating versus control adult ganglia (**Figure 1**) and in the surrounding blood sinus (**Figure 3**). Even in this restricted range, the mass spectra obtained at each location contain hundreds of peaks that might be of interest, and a criteron for selecting those that warrant further study needs to be formulated.

In our case, this criterion is the presence of a significant difference in the abundance (area under a peak) of the corresponding factor between the normal and the regenerating states. One way to analyze the massive amount of data produced by MSI and facilitate the identification of *m/z* values worthy of further study is to subject MSI datasets to PCA and clustering. When applied to the regenerating leech adult ganglia and blood sinus datasets, this analysis yielded two interesting results:

(1) A heterogeneous distribution favoring the presence and abundance of some of the clusters in the region of the ganglion proximal to the mechanical trauma (nerve crush), which we interpret as evidence for the accumulation of factors that are required by the process of regeneration in the damaged area.

(2) An apparent migration of blood molecular components into the CNS from the adjacent sinus, as denoted by the changes in cluster localization, indicating that blood-born peptides/proteins also migrate towards the site of damage.

Moreover, correlation between non targeted (MALDI imaging mass spectrometry) and targeted (DD-HPLC coupled to antimicrobial and neurotrophic tests) strategies showed that two of the 6 identified peptides (*m/z* 12,721, 13,684) corresponding to neurohemerytrin isoforms have been localized in the leech ganglia by MALDI-MSI. However, from the 21 detected by MALDI-MSI in the whole of ganglion, only four have been identified by DD-HPLC and bottom-up strategies. This last point show that it is now important to develop common experimental procedures and to create some new software [10] allowing such correlation between MALDI-MSI studies coupled to PCA and classical peptidomic/proteomic analyses. The power of MALDI-MSI is the ability to have access of a great number of peptides/proteins in non-targeted approach like classical proteomic, but establishing the spatial localization of the molecules in the tissues and getting access to the dynamic of the proteome in the organ.

While many of these peptides/proteins are probably housekeeping and general maintenance molecules that are required to reconstruct the damaged tissues, we expect that some will be specialized signals, neurotrophic and guidance factors, and neuro-specific molecules that are key to the re-establishment of a unique network of connections. It is the latter that we are particularly interested in identifying among the many *m/z* values present in the dataset.

Our data confirm the presence, and in some cases the up- or down-regulation, of different functional groups of proteins in the regenerating adult leech brain that are also expressed at significant levels in the embryonic CNS (**Tables 1** **–** [Table pone-0018359-t002] **3**; [3], [7], [9]). Among these are proteins implicated in cytoskeletal remodeling, including the Intermediate Filament (IF) proteins Gliarin and Macrolin [54] and the actin-binding protein Filamin [26]. Both ifs contain the coiled-coil rod domain typical of the superfamily of IF proteins flanked by unique N- and C-terminal domains, but Gliarin is found in all glial cells, including macro- and microglial cells [9], [54], whereas Macrolin is expressed in only a single pair of giant connective glial cells [54]. Interestingly, the *m/z* 2475 ion we selected for further study because it is present in embryonic ganglia and up-regulated in the adult CNS following injury (**Figure 2**, **Figure S1**), has been recently identified as a new member of the IFs, *Hm*IF4 [10]. In contrast to Gliarin and Macrolin, *Hm*IF4 appears to be highly expressed in neurons [10]. Filamin, with two calponin homology domains and 35 filamin/ABP-repeat domains, has been implicated along with Tractin in muscle development and nerve formation [26].

Another interesting functional group of proteins identified in this proteomic study as potentially involved in both neural development and regeneration is comprised of several neural members of the Ig superfamily (IgSF), specifically Tractin, Hillarin and the receptor tyrosine phosphatase *Hm*LAR2. Related to the last of these, we have recently reported that *Hm*LAR1, a sibling RPTP of *Hm*LAR2, is upregulated in specific neurons in response to a nerve crush, and that the regeneration of severed axonal projections is significantly impaired when RNAi is used to block this upregulation [29] Tractin and LeechCAM have been implicated in neurite outgrowth in the course of neurogenesis by Johansen, Zipser and collaborators [24], [26], [27], [55], [56]. Tractin is widely expressed and is differentially glycosylated in sets and subsets of peripheral sensory neurons that form specific fascicles in the central nervous system. Additional proteins identified in this screen that appear to be involved in neural regeneration include several previously identified in leech brain, including Netrin [28], Hillarin [57], and Lena (leech homolog of Enabled) [58]. Hillarin is localized to the axon hillock of leech neurons and affects cell and axonal cytokinesis through its interactions with septins [25], [59]. Lena, a cytosolic protein implicated in actin-based cell motility [58] has been shown to associate in the leech with the *Hm*LAR receptors, whose ectodomains are thought to promote an adhesive interaction that enhances neuronal sprouting [60].

An interesting and somewhat unexpected among our observations is the adult expression of the homeobox gene LOX2, which in the embryo is expressed in 25–30 pairs of neurons repeated in the posterior two-thirds of the midbody ganglia [61], [62]. Possibly this transcription factor regulates a set of specific growth responses that are triggered by neuronal damage, a hypothesis that has not been tested experimentally at this time.

Of particular novelty is the dynamic expression of three antimicrobial peptides in leech brain (**Figure 4** **,** **Table 2**) as a result of mechanical injury: *Hm*-neuromacin, *Hm-*lumbricin [14], and the novel one, *Hm*AMP3. These antimicrobial peptides produced by neurons and microglia have been recently shown by our group to promote the regeneration of neurites in axotomized leech CNS [14], indicating that they have multiple functional roles in the CNS. Moreover, other immune factors appear to also participate in the neuroregenerative process. We recently demonstrated that several leech CNS immune factors identified here (**Table 1**), such as the cytokines related to EMAP-II [11] and IL-16 [12] as well as the complement factor C1q [13], exert chemotactic effects towards leech microglia. In mammals, C1q is known to be synthesized and released by activated microglia in order to maintain and balance microglial activation in diseased CNS tissue [63].

Neuroinflammation is another key aspect of the neuroregenerative process. Molecules related to cyclophilin, ERp60 [9] in conjunction with cytokines like those related to IL-16, IL-17 and IL-25, are implicated in the control of inflammation [64]–[68] and the presence of such molecules in leech CNS [12], [18] after trauma suggests a generality and convergence of such a biological phenomena at early stage of the regeneration process. Neuroinflammation seems to be necessary for initiating microglial activation [69], [70], but it needs to be then controlled in order to block the apoptotic loss of neurons which may occur following excessive brain inflamation. This could be mediated by specific lipids that participate in the regulation of neuroinflammation, including cannabinoids and omega lipids [16], [19]. As we observed through *in vivo* and *in vitro* experiments, triacyl-sn-glycerols (C10, C14, C16), and omega lipids are synthetized as an early response to the lesion, in conjunction with endocannabinoids (AEA and 2-AG). These factors act as neuroimmunomodulators [71] and in the limited regeneration [72] in the mammalian spinal cord. The fatty acids are the targets of cerebral lipoxygenases that release very powerful anti-inflammatory factors, such as the neuroprotectins or the resolvins. In the series ω6, arachidonic acid is produced by all cells from the action of phospholipases and is the precursor of several neuroprotective agents, such as the endocannabinoids, which are synthesized in the brain and have anti-inflammatory properties. The major lipid in the ω9 series is oleic acid (C18:1), which also protects the nervous system by blocking the resulting inflammation after excessive stimulation (exitotoxicity). Sulfatides and gangliosides constitute another class of lipids preserving the central nervous system in vertebrates. Some studies have shown that their diminution at the cerebral level is directly associated with the manifestation of neurodegenerative conditions such as are observed in Alzheimer's disease [73]–[75]. In our context, stearic acid and phosphatidylinositol, as well the mono-unsaturated omega-9 fatty acid (oleic acid) are produced early after trauma and each show a specific pattern of expression, spatially and temporally. At the level of the trauma, oleic acid and phosphatidylinositol migrated from connective to neurons (packet cell) whereas stearic acid accumulate in connective and less in ganglion. This can be explained by the fact that oleic acid regulates inflammation at the level of neurons [76] whereas stearic acid seems to play a role in tissue repair and plasticity, near the lesionned connective [77], [78].

We propose that these data can be thought of together through the following model. Cannabinoids are more implicated at later stages of the regeneration/repair process. In fact, the neurite outgrowth tests showed that, among them, AEA is more important in scar formation whereas 2-AG appears to be more involved in axon extension. Cannabinoids are part of the regeneration process along with peptides and proteins, and all need to be taken into account together to achieve a deeper understanding of the whole of the biological process. In addition, microglial cells, in conjunction with neurons and blood cells are able to regulate neural inflammation very quickly and to stimulate neurite outgrowth, also with the release of cannabinoids, which at later stages appear to act along gangliosides in apoptosis regulation, neurite outgrowth and the release of axonal guidance factors. In the same time window, 3 hours after lesion, embryonic factors re-expression possibly occurs through homeobox gene activation, axon guidance and neurotrophic factors released. This is also linked to a close interaction between cells and the implication of intermediate filaments and cell-matrix interactions performing a net where neurites are able to sprout and receive some positive and negative factors like neuroregulins (erb-2 like factors, Cuvillier-Hot, unpublished data), inhibitors of NOGO and inhibitory factors.

In summary, proteomic and lipidomic approaches were employed to profile and identify different lipids and proteins in leech embryos as well as normal and regenerating adult leech nervous system. These are proposed as strong candidates for important roles in the mechanisms of neural regeneration (**Table 4**). The overlap between these profiles observed in regeneration in response to physical damage and in the neuroimmune response to bacterial insult suggests that these complex dynamic processes, involving many different types of cells and mediators, have much in common. Moreover, the overlap between molecular profiles observed in neural development and adult regeneration suggests that a significant recapitulation of neurogenic programs is present in the course of regeneration. The data presented here is only a beginning, but it already identifies similarities between the molecular underpinnings of invertebrate and vertebrate responses to trauma, similarities that can be exploited in furthering our understanding of the reasons for the limited capacity to regenerate neurites in the mammalian CNS.

## Materials and Methods

### Reagents

Alpha-cyano-4-hydroxycinnamic acid (CHCA), Sinapinic acid (SA), Trifluoracetic acid (TFA), Formic acid, Hydrochloric acid (HCl), Acetonitrile (AcN), Acetone, Ethanol (EtOH), Methanol (MeOH), Water CHROMASOLV PLUS for HPLC (H_2_O), Chloroform (CHCl_3_), Aniline (ANI), 2-Arachidonyl glycerol (2-AG), Lithium chloride, Ammonium bicarbonate (NH_4_HCO_3_) were purchased from Sigma-Aldrich (Saint-Quentin-Fallavier, France). Anandamide (AEA) was obtained from Merck Chemicals (Nottingham, UK). Sequencing grade modified trypsin, porcine enzyme was from Promega (Charbonnières-les-Bains, France).

### Animals

Leech embryos (E12 stages) and adults used in these experiments were obtained from *Hirudo medicinalis/Hirudo verbana* colonies maintained in our laboratories. Prior to use, embryos were removed from their cocoons and kept in artificial spring water (0.5 g/l Instant Ocean, Aquarium Systems) at 22°C, and staged according to the criteria of Fernandez and Stent [79]. At this temperature, day 0 (E0) is defined as the day of cocoon deposition and day 30 (E30) as the day of emergence of the juvenile animal from the cocoon.

### Sampling

All adult medicinal leeches sp. were purchased from Ricarimpex (Eysines, France). After arrival, leeches were kept without further feeding in artificial pond water until dissection. To produce controlled CNS lesions,following the procedure of Nicholls et al. [80], animals were pinned ventral-side up and small windows opened along the ventral midline in order to gain access to and partially cut the interganglionic connectives. After allowing a period for recovery and the establishment of a regenerative response (6 hours to 7 days), nerve cords were completely removed from the animals.

### Peptidomic and Proteomic Methods

#### Differential Display HPLC (DD-HPLC)

Nerve cords collected at different time of regeneration processes (20 nerve cords/time plot) were homogenized in phosphate buffered saline pH 7.5. Liquid was immediately centrifuged at 10,000 g at 4°C for 20 min and the supernatant was acidified by adjusting the pH to 3.9 with 1 M HCl. Centrifugation (10,000 g at 4°C for 20 min) was then used to clarify the supernatants, which were loaded onto Sep-Pak C18 Vac cartridges (Waters). Elution steps were performed with 2% and 60% AcN in H_2_O/0.01% TFA. The pre-purified fractions were then lyophilized, reconstituted in pure water and tested for neurite outgrowth activity. Only the 60% AcN eluted fractions were active and submitted to purification by reversed-phase high pressure liquid chromatography (RP-HPLC). All the purification steps were carried out on a Beckman™ Gold HPLC system. The 60% Sep-Pak fractions collected at each time plot of regeneration were subjected to RP-HPLC on a Sephasyl C18 column (250×4.1 mm, 218TP54 Vydac™). Elution was performed with a linear gradient of 2-62% AcN in acidified water over 90 min at a flow rate of 1 ml/min. Fractions corresponding to absorbance peaks were collected in polypropylene tubes, lyophilized, reconstituted in water and tested for neurite outgrowth activity tests (see below). Experiments were conducted 3-times.

For peptides identification, each fractions presenting a nerve sprouting activity were further loaded onto a C18 column (250×2.1 mm, 218TP52 Vydac™) with a gradient consisting in 2–25% AcN in acidified water for 10 min and 25–35% AcN for 40 min at a flow rate of 0.2 ml/min. Fractions were collected and treated as above. One additional step was performed on a narrow bore C18 reversed phase column (150×2 mm, Waters) at a flow rate of 0.2 ml/min using the AcN gradient described in the step 2. The purity assessment and the molecular mass determination of the peptides were carried out by Ultraflex II MALDI-TOF/TOF instrument (Bruker Daltonics, Bremen, Germany). N-terminal sequencing of the purified peptides was performed by automated Edman degradation on a pulse liquid automatic peptide sequenator (Beckman).

Peptides quantification has been carried out on lots of 20 nerve cords. Each lot was first weighed and then was submitted to the same procedures before being subjected to the Sep-Pak prepurification. Bradford quantification of each lot was performed before injection onto the RP-HPLC. Each collected peptide was tested on antimicrobial and neurotrophic tests activities and compared to internal standards [14], [15].

#### Neurotrophic activity assays

Dissected nerve cords were placed in 35 mm Petri dishes with 1 ml Leibovitz L-15 medium (Gibco, Invitrogen, USA) complemented with 2 mM L-glutamin, 0.6% glucose, 10 mM HEPES (complete medium), 10% Fetal bovine serum and antibiotics (100 UI/ml penicillin, 100 UI/ml streptomycin and 200 nM/ml gentamycin).

Neurite outgrowth in response to exposure to peptidic fractions purified by chromatography was measured in disocciated cultured leech neurons. Briefly, neurons mechanically dissociated from 3 nerve cords, were resuspended in 10 ml of L-15. Isolated neurons were split into the 96 wells of a culture plate and were then maintained at 20°C for one day before 10 µl of each RP-HPLC fraction were added to the 100 µl of culture medium. Neurite outgrowth of individual cells was quantified daily, up to 3 days after the addition of individual fractions to the culture, using the LEICA DMIRE2 inverted microscope (Leica Microsystems, Nanterre, France). Experiments have been conducted 5-times.

#### Antibacterial activity assays

After each purification step, antibacterial activity was monitored by a solid plate assay as described in previous studies [81]. The minimal inhibitory concentration and the minimal bactericidal concentration were determined according to the published method of Hancock, which is available at http://cmdr.ubc.ca/bobh/showmethod.php (method id_79).

#### Bottom-up strategy

Nerve cords collected at different times after lesioning (20 nerve cords/time plot) as well as embryos (E12 stages) were homogenized in phosphate buffered saline pH 7.5. Liquid was immediately centrifuged at 10,000 g at 4°C for 20 min and the supernatant was acidified by adjusting the pH to 3.9 with 1 M HCl. Centrifugation (10,000 g at 4°C for 20 min) was then used to clarify the supernatants, which were loaded onto Sep-Pak C18 Vac cartridges (Waters). Elution steps were performed with 2% and 80% AcN in 0.01% TFA. The 80% pre-purified fractions were then lyophilized, reconstituted in pure water and titrated in Bradford before submitted to trypsin digestion.

In order to achieve the trypsin digestions, samples (extracted proteins) were placed, after drying, on ice for 30 min in 50 µl of trypsin solution (0.02 mg/ml in 25 mM NH_4_HCO_3_). Digestion was then allowed to proceed overnight at 37°C. The reaction was stopped by the addition of either 50% AcN/1% TFA for further MALDI-MS analysis or 50% AcN/1% formic acid for further ESI-MS/MS analysis. Trypsin digests were then lyophilized in a SpeedVac concentrator. For nanoLC-MS/MS identification, peptides were re-dissolved in H_2_O/MeOH 0.1% formic acid (9∶1 v/v) after elution and evaporation.

NanoLC-ESI-IT MS and MS/MS analyses were performed on an ion trap mass spectrometer (LCQ deca XP plus, Thermo electron) equipped with a ESI ion source and on-line coupled to a nano HPLC system (Ultimate, Dionex). 0.5 µL of digest were injected with a Switchos Autosampler (Dionex corporation) and separation performed on a C18 silica bonded stationary phase (75 µm i.d., 150 mm long, 3 µm 100 Å pore size, Dionex). Samples were washed for 2 minutes at 10 µL/min with 100% mobile phase A (95% H_2_O, 5% ACN 0.1% formic acid). Peptides were then eluted using a linear gradient of 1%/min mobile phase B (ACN 80%, H_2_O 20%, formic acid 0.08%) for 70 minutes at a flow rate of 0.2 µL/min. The LCQ deca XP plus was operated in a data dependent MS/MS mode in which one MS full scan was followed by one MS/MS scan on the most abundant peptide ion. Collision energy was set to 35%. The heated capillary temperature and electrospray voltage were 160°C and 1.5 kV respectively.

Protein identification was performed with the Phenyx (Genebio) sequence query search program using the leech EST (http://genomes.ucsd.edu/leechmaster/transcriptome-paper/) and genomic (http://genome.jgi-psf.org/Helro1/Helro1.home.html) databases filtered for the taxonomy “*Hirudo medicinalis* and *Helobdella robusta*.” A tolerance of 1 Da for peptides in MS and 0.5 Da for MS/MS was used. Only peptides sequences with a MOWSE score higher than 32 (indicating significant homology or identity) and identified in several samples were considered. Methionine oxidation was defined as the variable modification.

#### MALDI imaging of Proteins

Leech nerve cords were included into gelatine 10% instead of OCT before sectioning. Thin 10 µm tissue sections were obtained using a cryostat Leica CM1510S (Leica Microsystems, Nanterre, France) and mounted onto ITO-coated conductive glass slides (Bruker Daltonics, Bremen, Germany). Several research groups have demonstred that the realization of chemical treatment on the tissue section can improve the quality of MS spectra. As described by Lemaire and co-workers, a washing step using chloroform allows to mainly remove phospholipids which can interfere in the spectra when analysing peptides [82]. Caprioli et al. showed the use of alcohol to fix the tissue and remove lipids and salts which can create a poor crystallization [83]. In this context, tissue sections were submitted to a washing step using cold EtOH 70% then cold EtOH 95% during 30 seconds for each bath followed by a washing step using chloroform during 30 seconds.

For matrix deposition, an ionic matrix solution containing 10 mg/ml of SA and 1 equivalent of ANI in AcN/TFA 0.1% (7∶3,v/v) was prepared. The matrix was deposited on leech tissue section with the automatic sprayer Imageprep (Bruker Daltonics, Bremen, Germany), which creates an aerosol by vibrational vaporization and provides high resolution images. For an optimal matrix coating the different steps *i.e.* spraying, incubation, drying and matrix-layer-thickness were monitored in real time. The method used here for SA/ANI deposition was a home-made method composed by an initialization phase in order to deposit a first layer of matrix on the slide followed by phases allowing a good co-crystallization of matrix crystals and analytes thanks to alternating cycles of partial and complete drying.

Image acquisitions were performed using an UltraFlex II MALDI-TOF/TOF instrument (Bruker Daltonics, Bremen, Germany) with a resolution of 50 µm in positive linear mode. Three hundred MALDI-MS spectra were acquired at each position in the mass range from 2000 to 20000 Da using a laser frequency of 50 Hz. Recording and reconstruction of the images were performed using FlexImaging 2.1 (Build 15) software (Bruker Daltonics, Bremen, Germany).

The normalization of MALDI images is a data processing that does not work on the single spectra, but on the dataset as a whole. The aim is to equalize the total ion count for all spectra. This is done to counter the effect of “hot spots” on the image. A hot spot could *e.g.* be a large matrix crystal, that was hit directly by the laser beam, resulting in a higher spectrum intensity at this point. The normalization has the disadvantage of artificially increasing the intensity of pure noise spectra. It is therefore possible to exclude noise spectra by defining an Y*mean*/Y*max cutoff*. Normalization is performed according to the calculation:
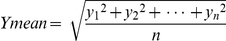
y  =  y value at a point of the spectrum and n  =  number of data points of the spectrum.

If Y*mean* < Threshold Factor * max. value of y-axis, then the spectrum will be normalized.

Each MALDI imaging experiments have been conducted 5 times.

### Lipidomics Methods

#### Endocannabinoid quantification

Endocannabinoid level measurement was performed in course of regeneration by MALDI-TOF mass spectrometry. 24 isolated nerve cords at 6 different time course of regeneration from 0 to 240 min were collected in physiological saline. Each set of 4 nerve cord sections (three ganglia and three connectives) was used to titrate the anandamide (AEA) and 2-arachidonoylglycerol (2-AG). Internally deuterated anandamide (4 µg/mL) and 2-arachidonoylglycerol (4 µg/mL) were added to each set before lipid extraction. From each set, the Folch method (chloroform/methanol (3∶1;v/v)) was performed for total lipid extraction. The chloroformic phase containing endogenous and deuterated endocannabinoids was harvested and submitted to a relative quantification.

For MALDI-TOF MS, lithium salt combined with DHB in acetone is known to be effective for determination of nonpolar long-chain lipids, hydrocarbons and polymers by MALDI [84]. Under these conditions, 3 mg of lithium chloride salt with 6 mg of 2,5-DHB were dissolved in 100% acetone. Lithiated matrix and lipid extracts (1∶1;v/v) were mixed and dropped onto a 384 stainless steel sample plate (Bruker Daltonics Bremen, Germany). Mass spectrometric analyses were performed in positive reflector mode with an UltraFlex II MALDI-TOF/TOF instrument (Bruker Daltonics, Bremen, Germany) equipped with a Smartbeam laser having a repetition rate up to 200 Hz and controlled by FlexControl 3.0 (Build184) software (Bruker Daltonics, Bremen, Germany). The spectra were treated with FlexAnalysis 3.0 (Build 96) software. Linearity of the mass spectrometry measurements was determined with endocannabinoid standards by measuring the ratio of their accumulated (8000 laser smartbeam shots) area spectra over the corresponding internal deuterated standard one. The measurements obtained for accumulated spectra showed linear responses within the referenced range. Relative quantification of 2-AG and AEA was performed by stable isotope dilution (2-AGd8 and AEAd8). The presence of 2-AG and AEA was determined by observing that their respective ions of *m/z* 385.29 and 354.29 were isolated and fragmented according the positive LIFT mode of the MALDI-TOF mass spectrometer and compared to their corresponding standards. Experiments were conducted 3-times.

#### Physiological activity of endocannabinoids

For neurite outgrowth assay, dissected nerve cords (three per condition) were pinned in a dish coated with silicone rubber (Sylgard 184) containing supplemented L-15 medium under sterile conditions [12]. To investigate the process of neurite outgrowth, one lateral connective nerve was cut in each nerve cord and drugs (AEA, capsacin, aravanil, capsazepin) were added to the culture medium. Controls consisted of the absence of drugs. Images of the cut ends were taken every 24 hours for 1 week (objective X5) using an inverted microscope (as described above) and analyzed using the Bioposition V3.0 software (developed on the Matrox MIL7.5 Base Library by Gilles Courtand, Centre Commun de Mesures Imagerie Cellulaire, University of Lille, France).

#### ToF-SIMS imaging

Leech nerve cords in course of regeneration (0, 6 hours, 24 hours as described above) were included into gelatine 10% instead of OCT before sectioning. Thin 10 µm tissue sections were obtained using a cryostat Leica CM1510S (Leica Microsystems, Nanterre, France) and mounted onto silicon wafer before analyzed by ToF-SIMS.

A standard commercial ToF-SIMS IV (Ion-Tof GmbH, Münster, Germany) reflectron-type ToF mass spectrometer was used for MSI experiments. The primary ion source was a bismuth liquid metal ion gun. Bi_3_^+^ cluster ions were selected. The ion column focusing mode ensured both a 1−2 µm beam focus and short pulse duration of less than 1 ns. Such short pulses are a prerequisite for high mass resolution, accurate mass measurements, and structure assignments. Because of the very low initial kinetic energy distribution of the secondary ions, the relationship between the time-of-flight and the square root of *m/z* is always linear over the whole mass range. The mass calibration was always internal and signals used for initial calibration were those of H^+^, H_2_^+^, H_3_^+^, C^+^, CH^+^, CH_2_^+^, and CH_3_^+^ for the positive ion mode and the signals of C^−^, CH^−^, C_2_^−^, and C_2_H^−^ for the negative ion mode. Signals of cholesterol were used for the positive ion mode calibration refinement, and for negative ion mode, fatty acid carboxylate ions were selected. Structure attributions or assignments of ion peaks were made according to the instrument resolution (*M*/Δ*M*  = 10^4^, full width half-maximum [fwhm], at *m/z* 500), accuracy, and the valence rule. A more detailed description can be found elsewhere [85]. Molecular images were recorded with a field of view of 500×500 µm^2^ and 256×256 pixels, giving a pixel size of 2×2 µm^2^. In these conditions, the fluence was always kept to 1.5×10^11^ ions.cm^−2^. A low energy electron flood gun is activated between two primary ions pulses in order to neutralize the sample surface with the minimum damage. The *m/z* value of the peak centroid, the maximal number of counts in a pixel (mc) and the total number of counts (tc) are written below each image. The color scales correspond to the [0, mc] intervals. The data acquisition and processing softwares were IonSpec and IonImage (ToF-SIMS software V4.1, Ion-Tof GmbH, Münster, Germany).

### Genomic and Transcriptomic Methods

Two TRPV sequences were identified through homology-based alignment between mammalian TRPV protein sequences and six-frame translations of contiguous DNA sequences (contigs) assembled from high-throughput short-read sequences. The draft genome contigs were obtained by assembling a total of 73 million paired-end reads and 56 million single reads, all of length 100. A separate, single animal was used to prepare DNA for sequencing libraries. Assembly was performed with EULER-SR, Velvet Optimizer, and Velvet [18], [48]–[50] Human, mouse and rat protein sequences for TRPV1-6 were downloaded from the Uniprot Database [86], [87] and subjected to alignment with contigs using the Tblastn algorithm [52]. (Note, rat TRPV3 is missing from the curated databases). Protein subsequences translated from aligned contig subsequences and the alignment coordinates were extracted from tabular blast output; corresponding DNA subsequences were extracted from contigs. Protein and DNA sequences were joined with custom perl scripts into putative *Hirudo* protein and their coding sequences (CDS). The *Hirud*o proteins were aligned with proteins downloaded from the *Helobdella* genome website [47] and expressed sequences from the *Hirudo* transcriptome [18] to obtain two *Helobdella* TRPV orthologs and confirm expression of TRPV2 in *Hirudo*. *Hirudo* and *Helobdella* proteins were aligned with the protein database at GenBank to confirm that TRPVs were the best matching proteins in human, and OSM1 proteins were the best matches in *C. elegans, D. melanogaster*, and other insects. TRPV and OSM proteins from leech, Human, mouse, rat, worm, and fly were multiply aligned with clustalw [88] and trees generated from distances output by clustalw.

## Supporting Information

Figure S1 **The** ***m/z*** **2475 ion is not expressed in control adult CNS segmental ganglia.** **A**. MALDI-MS average spectrum acquired from 9 sections of the non regenerating adult ganglion. The ion with *m/z* 2475 is not detected in control ganglion sections (labeled by the red cross). **B**. Distribution of the *m/z* 2475 ion in sections of the non regenerating adult ganglion. The insert shows a magnified image of the data for section 4, with the abundance of the ion color coded according to the color bar at right.
(TIF)

Figure S2 **Hierarchical clustering of spectra from 9 sections of a control ganglion and surrounding blood sinus.** **A**. Full dendrogram of all spectra in the ganglion dataset yields two main branches, colored red and green, that segregate into different domains in the images, (panel B). **B**. Reconstruction of selected dendrogram branches and corresponding images shows that the lower branch (green) corresponds with the blood cells (annulus around the central region) while the upper branch (red) corresponds to cells in the CNS region. In all sections, the blood sinus peptide profile (green) appears to be separate from the CNS peptide profile (red).
(TIF)

Figure S3 **Sequence alignment of proteins present during neurogenesis and regeneration.** The peptides obtained by shot-gun were balst against Hirudinae EST library using Blast-P.
(TIF)

Figure S4 **Leech nerve cord peptides and its differential distribution in crush direction using MALDI-MS imaging approach.** Each 10 µm thick section is spaced about 20 µm.
(TIF)

Figure S5 **Coding DNA and amino acid sequences for** ***Hirudo*** **TRPV1, TRPV2, and putative partial TRPV3 fragment.** DNA sequences were extracted from a draft genome assembly based on alignment with *Helobdella*, human, mouse and rat TRPV protein sequences. Protein sequences are translations of CDS. Exons missing from the draft annotation are designated with “n” (DNA) or “X” (amino acid). The TRPV3 fragment contains a well-conserved ankarin domain. All three predicted *Hirudo* TRPV's have best matches with TRPVs in mammals, insects, and worm.
(TIF)
