# Supplementary figures and images for: Multiple Changes in Peptide and Lipid Expression Associated with Regeneration in the Nervous System of the Medicinal Leech
Source: PLoS One. 2011 Apr 22;6(4):e18359. doi: 10.1371/journal.pone.0018359 (PMC3081291; doi:10.1371/journal.pone.0018359)

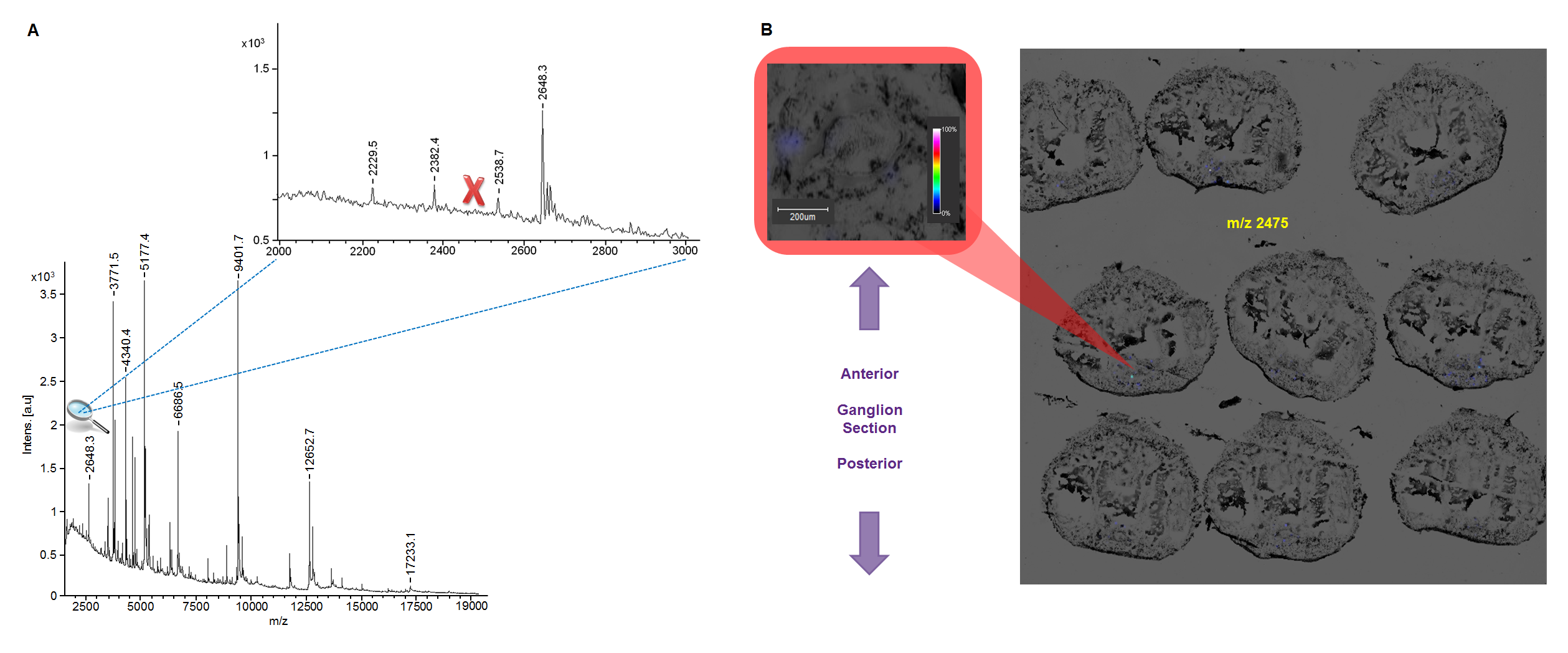

Supplement: Figure S1 — The m/z 2475 ion is not expressed in control adult CNS segmental ganglia. A. MALDI-MS average spectrum acquired from 9 sections of the non regenerating adult ganglion. The ion with m/z 2475 is not detected in control ganglion sections (labeled by the red cross). B. Distribution of the m/z 2475 ion in sections of the non regenerating adult ganglion. The insert shows a magnified image of the data for section 4, with the abundance of the ion color coded according to the color bar at right. (TIF) [file pone.0018359.s001.tif]

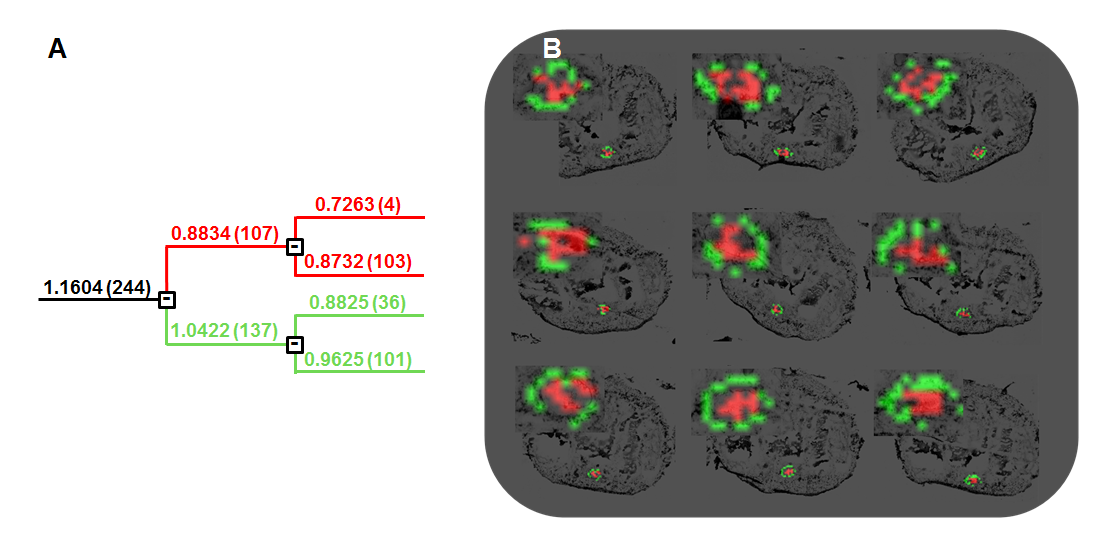

Supplement: Figure S2 — Hierarchical clustering of spectra from 9 sections of a control ganglion and surrounding blood sinus. A. Full dendrogram of all spectra in the ganglion dataset yields two main branches, colored red and green, that segregate into different domains in the images, (panel B). B. Reconstruction of selected dendrogram branches and corresponding images shows that the lower branch (green) corresponds with the blood cells (annulus around the central region) while the upper branch (red) corresponds to cells in the CNS region. In all sections, the blood sinus peptide profile (green) appears to be separate from the CNS peptide profile (red). (TIF) [file pone.0018359.s002.tif]

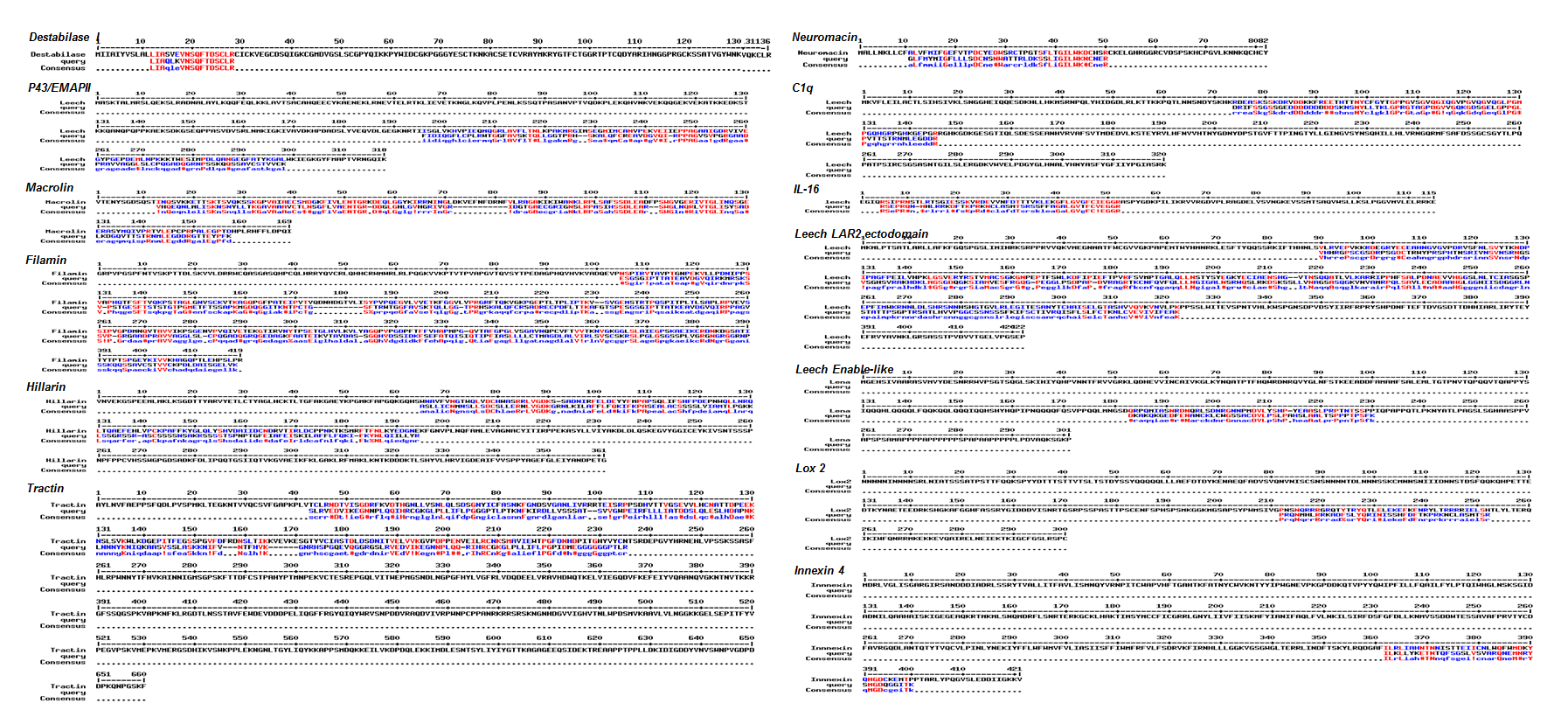

Supplement: Figure S3 — Sequence alignment of proteins present during neurogenesis and regeneration. The peptides obtained by shot-gun were balst against Hirudinae EST library using Blast-P. (TIF) [file pone.0018359.s003.tif]

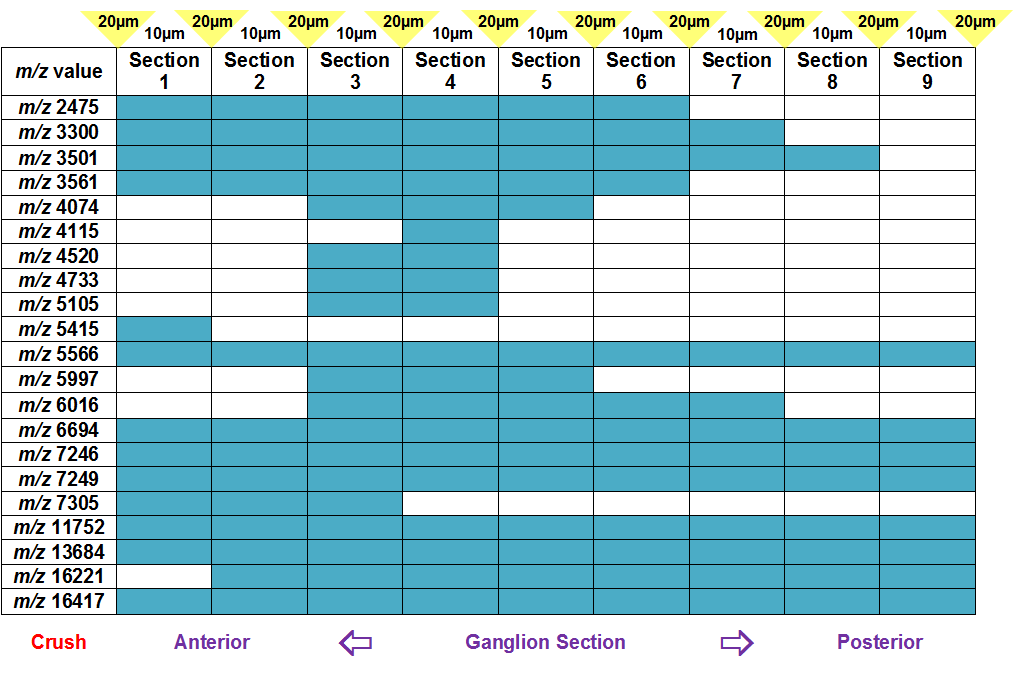

Supplement: Figure S4 — Leech nerve cord peptides and its differential distribution in crush direction using MALDI-MS imaging approach. Each 10 µm thick section is spaced about 20 µm. (TIF) [file pone.0018359.s004.tif]

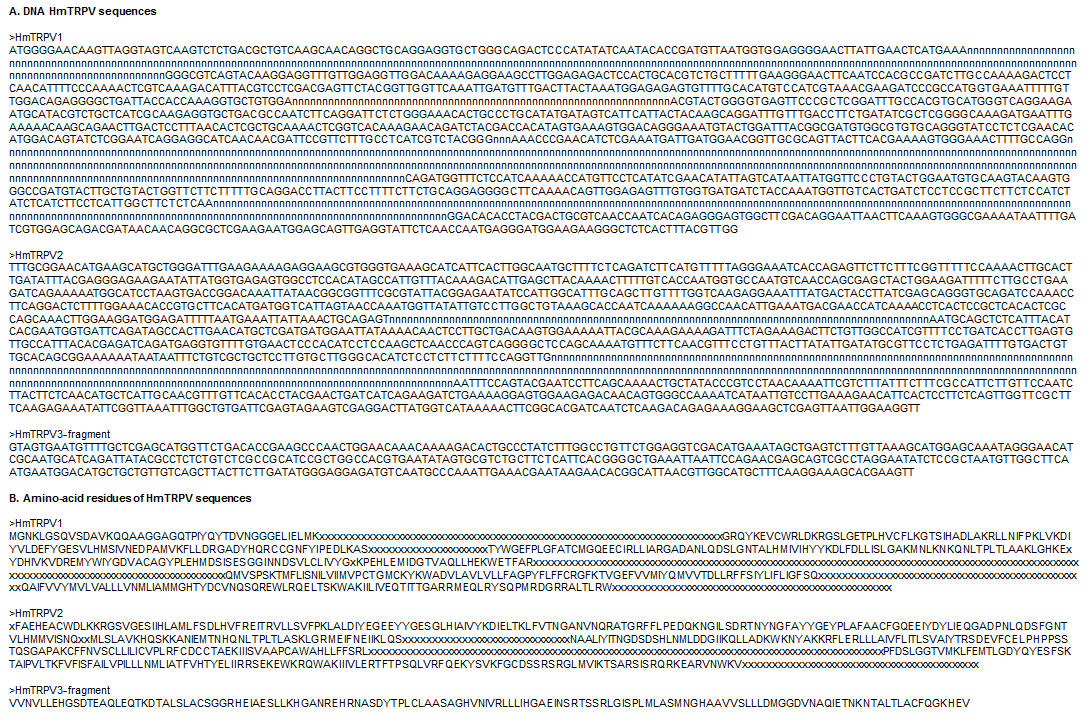

Supplement: Figure S5 — Coding DNA and amino acid sequences for Hirudo TRPV1, TRPV2, and putative partial TRPV3 fragment. DNA sequences were extracted from a draft genome assembly based on alignment with Helobdella, human, mouse and rat TRPV protein sequences. Protein sequences are translations of CDS. Exons missing from the draft annotation are designated with “n” (DNA) or “X” (amino acid). The TRPV3 fragment contains a well-conserved ankarin domain. All three predicted Hirudo TRPV's have best matches with TRPVs in mammals, insects, and worm. (TIF) [file pone.0018359.s005.tif]
